# DNMT1-mediated SPINT2 expression drives early senescence by suppressing c-Met signaling in human fibroblasts

**DOI:** 10.18632/aging.206303

**Published:** 2025-08-20

**Authors:** Min Seok Sim, Hae-Ok Byun, Seongki Min, Jiwon Hong, Su Bin Lim, Kyoung Sook Choi, Gyesoon Yoon

**Affiliations:** 1 Department of Biochemistry and Molecular Biology, Ajou University School of Medicine, Suwon 16499, Korea; 2 Inflamm-aging Translational Research Center, Ajou University Medical Center, Suwon 16499, Korea; 3 Department of Biomedical Sciences, Graduate School of Ajou University, Suwon 16499, Korea

**Keywords:** senescence, cellular senescence, aging, DNA methyltransferase 1 (DNMT1), serine protease inhibitor Kunitz type 2 (SPINT2)

## Abstract

Cellular senescence is a critical process involved in aging and related disorders, yet the molecular triggers of early senescence remain elusive. Here, we identify DNA methyltransferase 1 (DNMT1) downregulation as a key trigger of early senescence and establish serine protease inhibitor Kunitz type 2 (SPINT2) as its critical downstream effector. Using replicative and oxidative stress-induced senescence models of primary human diploid fibroblast, we observed persistent upregulation of SPINT2 and inverse downregulation of DNMT1, preceding senescence-associated β-galactosidase activity, a conventional senescence marker. Pharmacological inhibition and siRNA-mediated knockdown of DNMT1 significantly increased SPINT2 expression and induced senescence, showing mitigated effects by SPINT2 knockdown. Furthermore, SPINT2 overexpression alone induced senescence. Methylation-specific sequencing identified four CpG sites in SPINT2 promoter, that became hypomethylated at early transition of senescence and upon DNMT1 suppression. Functional analyses revealed that DNMT1-mediated SPINT2 expression induced c-Met inhibition, triggering senescence. Transcriptomic profiling identified 17 commonly deregulated c-Met signaling genes in both senescence models, with COL27A1, STAM2, and CBL validated as key downstream targets of SPINT2/c-Met signaling. These findings establish DNMT1-mediated SPINT2 upregulation as a novel epigenetic mechanism driving senescence initiation via c-Met inhibition, providing insights into the early stage of senescence and potential therapeutic targets for aging-related diseases.

## INTRODUCTION

Cellular senescence is characterized by an irreversible loss of replicative capacity and distinct phenotypic changes, including enlarged cellular morphology, increased reactive oxygen species (ROS) production, senescence-associated β-galactosidase activity (SA-β-gal), and senescence-associated secretory phenotypes [1–3]. These changes occur progressively and are accompanied by dynamic shifts in gene expression [3–6]. Most studies on senescence focus on fully senescent cells, partly due to the difficulty in detecting early-stage senescent cells. SA-β-gal, a widely used marker of senescence [7], is only expressed after a cell has fully transitioned into senescence [4], limiting its use for studying senescence initiation. Understanding the molecular regulators that drive early senescence is crucial for developing therapeutic strategies to modulate cellular aging and senescence-associated diseases. To address this gap, we utilized two time-series models of human diploid fibroblasts (HDFs): replicative senescence (RS) and oxidative stress-induced senescence (OSIS) [8–11]. These models allow the identification of key regulators of senescence prior to SA-β-gal activation.

DNA methylation plays a fundamental role in regulating gene expression and is controlled by two classes of DNA methylation activities: *de novo* methylation (mediated by DNMT3A and DNMT3B) and maintenance methylation (primarily mediated by DNMT1) [12, 13]. During cell replication, DNMT1 ensures the accurate transfer of DNA methylation marks to newly replicated DNA strands, preserving gene expression patterns and cellular identity in daughter cells [14, 15]. DNMT1 targets specific genomic regions known as CpG islands, where it modulates gene expression by methylating cytosine residues [16]. Recent studies have revealed a compelling link between suppressed DNMT1 activity and cellular senescence [9, 17, 18]. DNMT1 inhibition disrupts the faithful transmission of DNA methylation patterns during cell division, leading to epigenetic alterations that trigger senescence-associated gene expression programs [19, 20]. Additionally, DNMT1 expression is commonly downregulated from the initial stages before the onset of SA-β-gal activity in both replicative senescence (RS) and oxidative stress-induced senescence (OSIS) models of human diploid fibroblasts (HDF) [9], implicating its primary involvement in initiating senescence-associated gene reprogramming and expressing specific downstream target genes to trigger senescence.

Serine protease inhibitor Kunitz type 2 (SPINT2), a transmembrane protein with two extracellular Kunitz domains, inhibits the activities of diverse serine proteases, including hepatocyte growth factor activator (HGFA) and matriptase [21–23]. HGFA cleaves *de novo* single-chain structure of hepatocyte growth factor (HGF) to convert it into active form [24]. Eventually, SPINT2 blocks HGF activation and subsequent c-Met signaling pathways which modulate various cellular processes, including cell proliferation and motility [25, 26]. Interestingly, c-Met suppression induces senescence-like phenotypes in glioblastoma cell, supporting the role of c-Met as a marker of senescence [27]. These findings suggest that *SPINT2*-mediated inhibition of c-Met may be a key for the early transition of cellular senescence.

In this study, we show that *SPINT2* upregulation and *DNMT1* downregulation occur in an inverse manner during early senescence, preceding SA-β-gal activation. We further demonstrate that DNMT1 regulates *SPINT2* through DNA methylation at specific CpG sites, leading to *SPINT2*-mediated inhibition of c-Met signaling. These findings reveal a novel epigenetic mechanism in senescence and identify potential therapeutic targets for senescence-associated disorders.

## RESULTS

### Inverse regulation of SPINT2 and DNMT1 during senescent process of primary HDF

**Figure 1:**
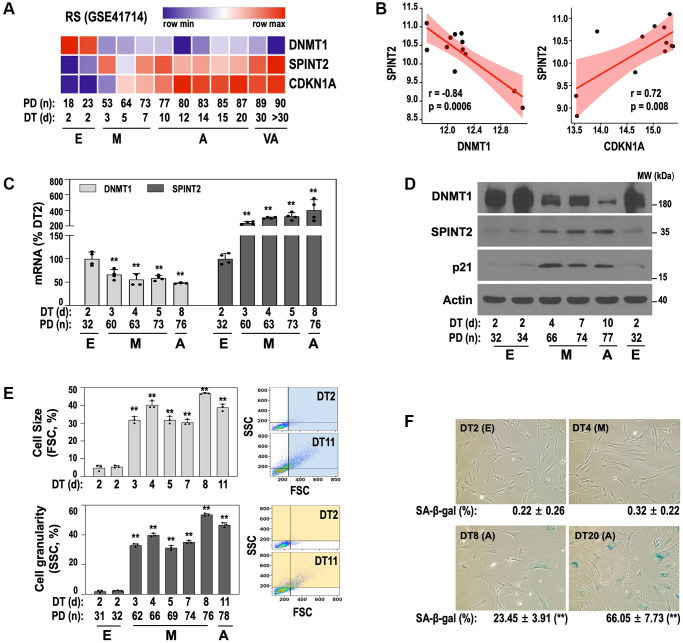
**An inverse correlation between DNMT1 and SPINT2 emerges at the early transition (E-to-M) to senescence in RS of primary HDF.** (**A**, **B**) Heatmap (**A**) and correlation plot (**B**) depicting DNMT1 and SPINT2 expression dynamics throughout RS process. Transcriptomic data were obtained from our previously established time-series RS model of primary HDF (GSE41714). The different stages of RS—early (E), middle (M), advanced (A), and very advanced (VA)—are indicated. (**C**–**E**) RS of primary HDFs was induced as described in the 'Experimental Procedures' section. (**C**) qRT-PCR analysis of *DNMT1* and *SPINT2* mRNA expression. ^**^p < 0.01 vs. DT2 by Student’s *t*-test. (**D**) Western blot analysis of DNMT1, SPINT2, and p21 protein expression. (**E**) Cell size (upper) and granularity (lower) were assessed by side scatter (SSC) and forward scatter (FSC), respectively, using flow cytometry. ^**^p < 0.01 vs. DT2 by Student’s *t*-test. Bar graphs represent mean ± standard deviation (SD) from three independent cultures. Representative SSC and FSC patterns of DT2 and DT11 cells are shown in the right panel. (**F**) SA-β-gal assay was performed at each stage. Representative images are shown and quantifications of SA-β-gal-positive cells are presented below each image. ^**^p < 0.01 vs. DT2 by Student’s *t*-test.

To investigate the role of *SPINT2* and its relationship with *DNMT1* in cellular senescence, we analyzed time-series transcriptome data from RS process of HDF (GSE41714) previously generated by our group [4]. It is noteworthy that development of this RS model took over a year and the model delineated four distinct stages of senescence progression—early (E), middle (M), advanced (A), and very advanced (VA)—each characterized by unique gene expression patterns. Our analysis revealed a continuous increase in *SPINT2* expression from DT3 (the starting point of M stage) to the end VA stage while DNMT1 declined during the same period (Figure 1A). Additionally, *CDKN1A* (p21), a senescence marker gene [3], exhibited a progressive increase, with its induction slightly lagging behind *SPINT2* expression (Figure 1A). Pearson correlation analysis confirmed a strong inverse correlation between *SPINT2* and *DNMT1* and a direct correlation between *SPINT2* and *CDKN1A* (Figure 1B). These deregulated expression levels were further validated at both the mRNA and protein levels (Figure 1C, 1D). Notably, M-stage cells displayed increased cell size and granularity (Figure 1E), which is indicative of early senescence phenotypes [4], but did not yet exhibit increased SA-β-gal activity (Figure 1F), a conventional senescence marker.

**Figure 2:**
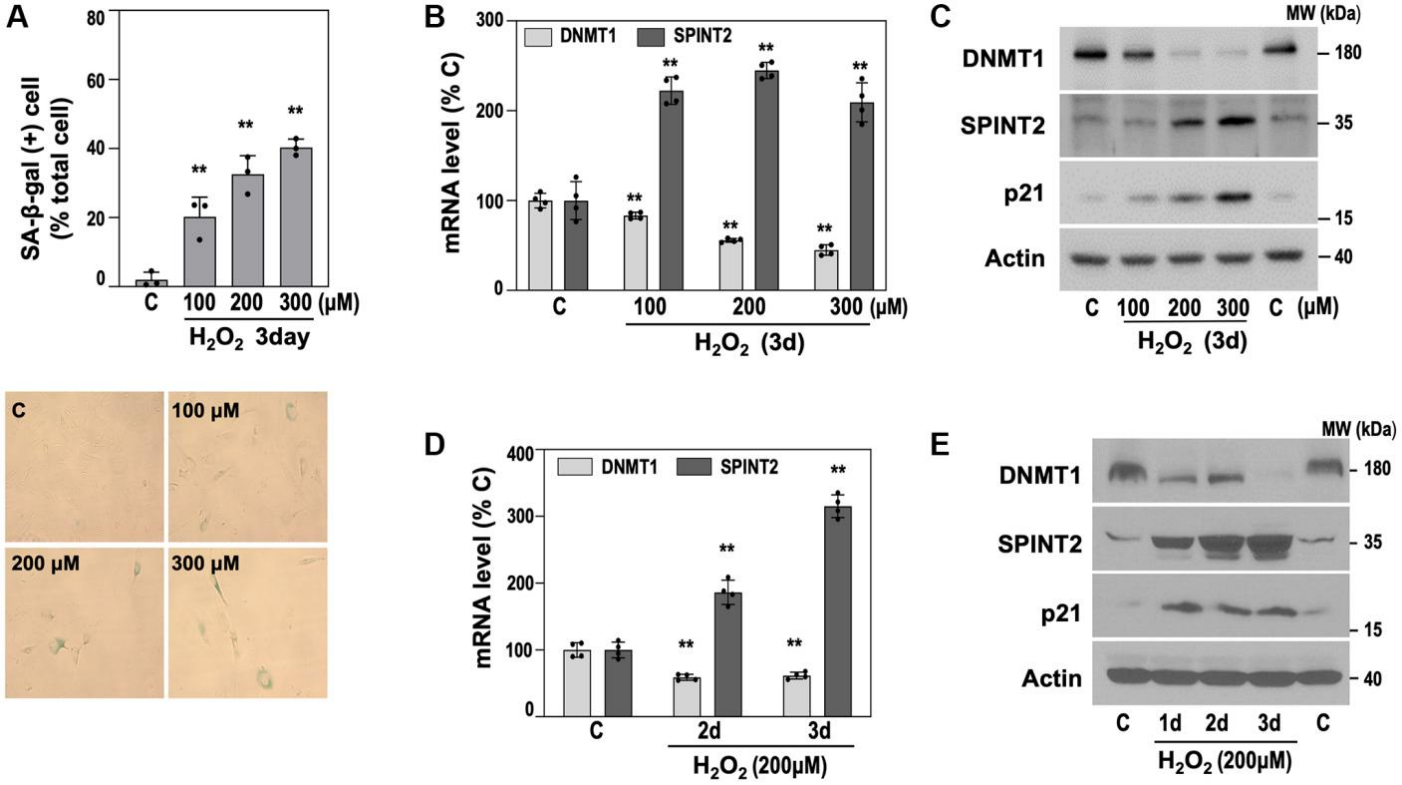
**The inverse relationship between DNMT1 and SPINT2 expression is also observed in the OSIS model of primary HDF.** (**A**–**C**) OSIS was induced by exposing primary HDFs (DT2) to the indicated concentrations of H_2_O_2_ for 3 days. (**A**) SA-β-gal activity was quantified (upper panel), and representative staining images are shown (lower panel). ^**^p < 0.01 vs. HDF DT2 control (C) by Student’s *t*-test. (**B**) qRT-PCR analysis of *DNMT1* and *SPINT2* mRNA levels. ^**^p < 0.01 vs. C by Student’s *t*-test. (**C**) Western blot analysis of DNMT1, SPINT2, and p21 protein expression. (**D**, **E**) HDFs (DT2) were treated with 200 μM H_2_O_2_ for the indicated time points. (**D**) qRT-PCR analysis of *DNMT1* and *SPINT2* mRNA levels. ^**^p < 0.01 vs. control (**C**) by Student’s *t*-test. (**E**) Western blot analysis of DNMT1, SPINT2, and p21 protein expression.

To determine whether this inverse relationship between *SPINT2* and *DNMT1* is a general feature of cellular senescence, we established an OSIS model by exposing primary HDFs (DT2) to subcytotoxic doses of H_2_O_2_ (100–300 μM). While RS takes over a year to develop, OSIS occurs within three days (Figure 2A). Cells treated with H_2_O_2_ exhibited senescence phenotypes, including increased SA-β-gal activity (Figure 2A) and a dose-dependent upregulation of p21 protein expression (Figure 2C). In this OSIS model, both *SPINT2* mRNA and protein levels significantly increased, whereas *DNMT1* expression decreased (Figure 2B, 2C), mirroring the pattern observed in RS model. A time-course analysis with 200 μM H_2_O_2_ further confirmed the progressive inverse regulation of *SPINT2* and *DNMT1* (Figure 2D, 2E). These findings demonstrate that the inverse regulation of DNMT1 and SPINT2 is a shared molecular event in the early onset of HDF senescence.

### DNMT1 suppression triggers SPINT2 induction and HDF senescence

**Figure 3:**
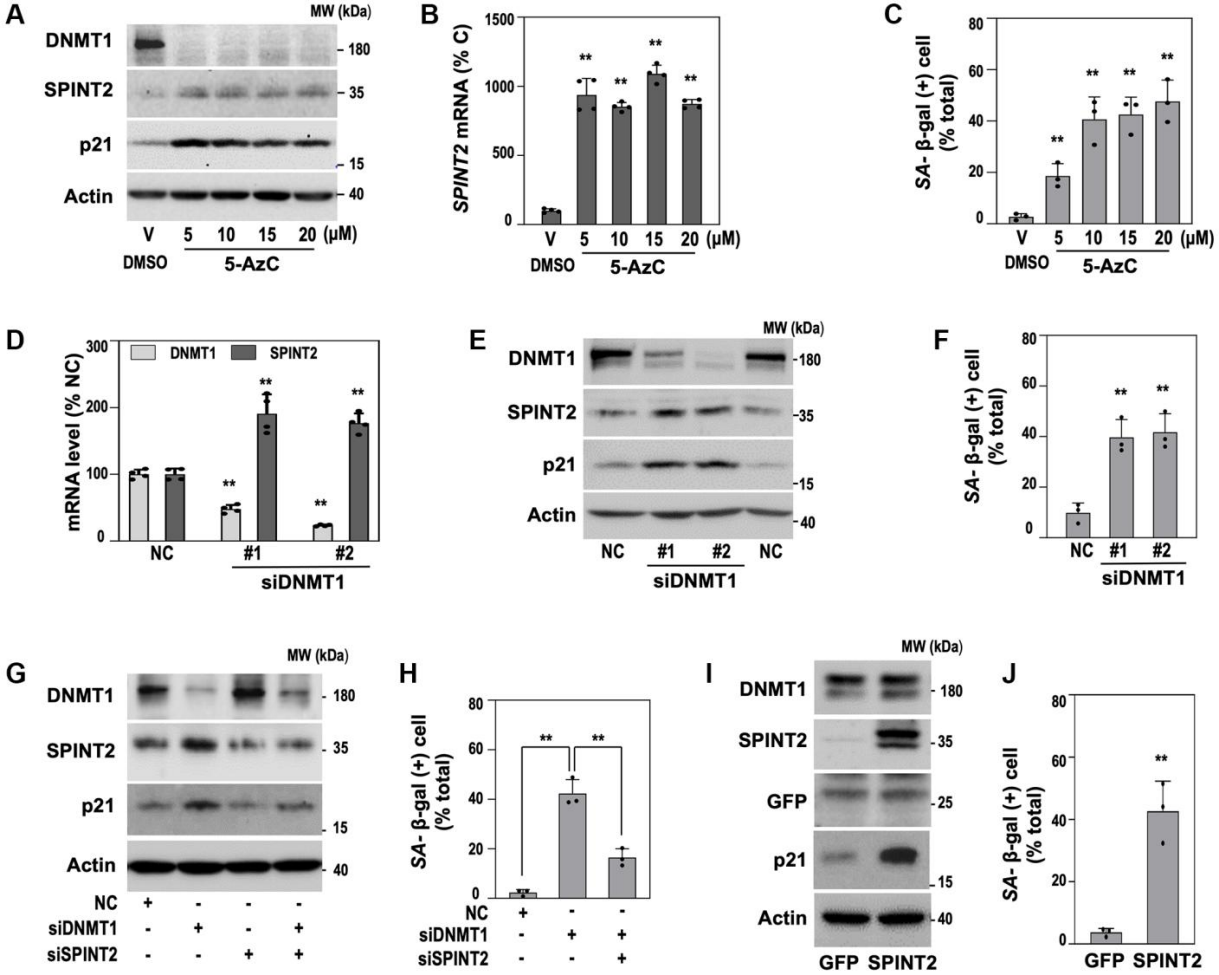
**DNMT1 inhibition induces senescence via SPINT2 upregulation.** (**A**–**C**) HDFs (DT2) were treated with indicated concentrations of 5-AzC for 3 days. (**A**) Western blot analysis of DNMT1, SPINT2, and p21 protein expression. (**B**) qRT-PCR analysis of *SPINT2* mRNA expression. ^**^p < 0.01 vs. DMSO control (V) by Student's *t*-test. (**C**) Quantification of SA-β-gal activity ^**^p < 0.01 vs. V by Student’s *t*-test. (**D**–**H**) HDFs (DT2) were transfected with siRNAs targeting the indicated genes for 4 days. (**D**) qRT-PCR analysis of *DNMT1* and *SPINT2* mRNA levels. ^**^p < 0.01 vs. negative control siRNA (NC) by Student’s *t*-test. (**E**) Western blot analysis of DNMT1, SPINT2, and p21 protein expression. (**F**) Quantification of SA-β-gal activity. ^**^p < 0.01 vs. NC by Student’s *t*-test. (**G**) Western blot analysis of DNMT1, SPINT2, and p21 protein expression. (**H**) Quantification of SA-β-gal activity following co-transfection ^**^p < 0.01 vs. NC by Student’s *t*-test. (**I**, **J**) HDFs (DT2) were infected with the recombinant lentivirus encoding *SPINT2* cDNA for 4 days. GFP-encoded recombinant lentivirus was used as a control. (**I**) Western blots analysis. (**J**) Quantification of SA-β-gal activity. ^**^p < 0.01 vs. GFP by Student’s *t*-test.

To investigate whether *DNMT1* directly regulates *SPINT2*, we treated young HDFs (DT2) with 5-aza-2′-deoxycytidine (5-AzC), a DNA methylation inhibitor [28]. Consistent with previous reports [28], 5-AzC treatment significantly reduced DNMT1 protein levels (Figure 3A, top) while increasing *SPINT2* expression at both mRNA and protein levels (Figure 3A, 3B). These molecular changes were accompanied by senescence phenotypes, including p21 induction (Figure 3A) and increased SA-β-gal activity (Figure 3C). To confirm that *DNMT1* suppression directly induces *SPINT2*, we performed siRNA-mediated knockdown of *DNMT1*. This significantly upregulated *SPINT2* at both the mRNA and protein levels (Figure 3D, 3E) and induced senescence phenotypes (Figure 3F). Importantly, co-transfection of *SPINT2* siRNA in *DNMT1*-silenced cells significantly reduced senescence phenotypes (Figure 3G, 3H), indicating that *SPINT2* is a key downstream mediator of *DNMT1* suppression-induced senescence. Furthermore, SPINT2 overexpression alone was sufficient to induce senescence, even in the absence of *DNMT1* suppression (Figure 3I, 3J), suggesting that *SPINT2* acts as an independent pro-senescence factor. These results confirm that *DNMT1* downregulation is a critical upstream regulator of SPINT2-mediated senescence.

### SPINT2 is transcriptionally regulated by DNMT1-mediated DNA methylation

**Figure 4:**
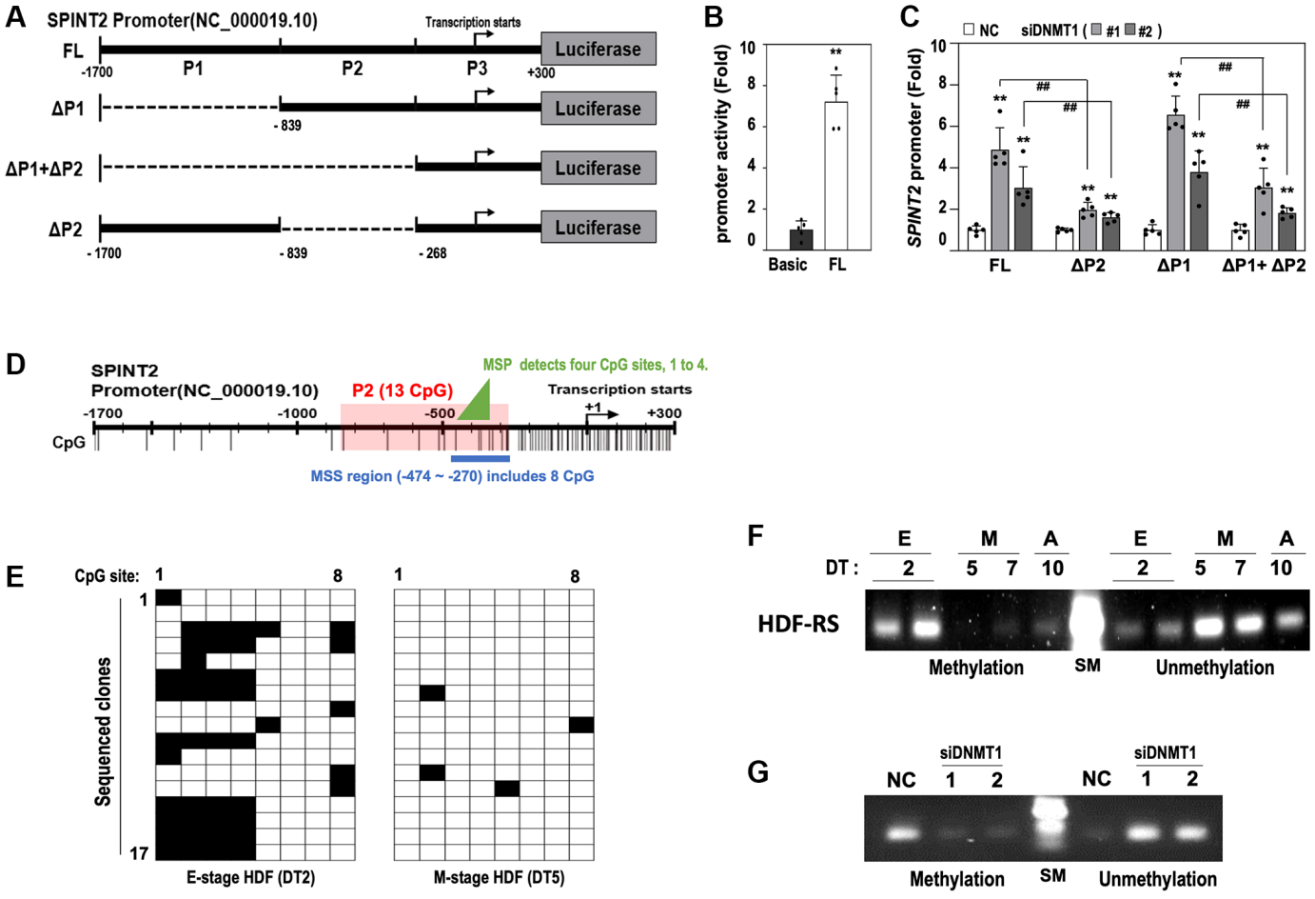
**DNMT1 regulates SPINT2 expression via promoter methylation.** (**A**) Schematic representation of *SPINT2* promoter constructs used in the luciferase reporter assay, with indicated deletion sites (FL, full-length of −1700 to +300; ΔP1, deletion of −1700 to −839; ΔP1+ΔP2, deletion of −1700 to −268; ΔP2, deletion of −839 to −268). (**B**) HDFs (DT2) were transfected with pGL4-basic or pGL4-SPINT2 promoter reporter constructs for 4 days, followed by luciferase assay. (**C**) HDFs (DT2) were transfected with siRNAs targeting negative control (NC) or *DNMT1* (siDNMT1) for 24 hours, then transfected with *SPINT2* promoter reporter constructs for 2 days. Cell lysates were analyzed for luciferase activity. (**D**) Schematic representation of CpG islands in the *SPINT2* promoter and selected regions used for DNA methylation-specific sequencing (MSS) and methylation-specific PCR (MSP) analyses. (**E**) MSS analysis of DNA clones from DT2 (early-stage) and DT5 (middle-stage) HDFs. Methylated CpG sites are shown in black, and unmethylated sites in white. Seventeen DNA clones were sequenced per group. (**F**) Methylation-specific PCR (MSP) analysis of the four CpG methylation hot spots (−445, −366, −363, and −358) identified by MSS. SM indicates DNA size marker. (**G**) HDFs (DT2) were transfected with siRNAs for negative control (NC) or DNMT1 (siDNMT1) for 3 days and then subjected to MSP analysis against the 4 CpG methylation hot spots.

To further explore the transcriptional regulation of *SPINT2* by *DNMT1*, we constructed a *SPINT2* promoter reporter plasmid containing a full-length promoter region (FL, −1700 to +300) and generated deletion mutants (ΔP1, ΔP1+ΔP2, and ΔP2) as shown in Figure 4A. Transfection of the FL construct into young HDFs (DT2) resulted in a significant increase in promoter activity compared to the basic reporter plasmid, confirming that this region contains essential regulatory elements (Figure 4B). Upon DNMT1 knockdown, the *SPINT2* promoter activity of both the FL and ΔP1 constructs increased significantly while ΔP2 construct showed minor increase (Figure 4C). Additionally, ΔP1+ΔP2 promoter activity was significantly lower than ΔP1, but slightly higher than ΔP2 construct (Figure 4C), indicating that the P2 region plays a critical role in *DNMT1*-mediated *SPINT2* transcription, whereas the P1 region may contain a negative regulatory element. Figure 4D illustrates the positions of CpG sites within the FL *SPINT2* promoter region. The P2 region contains thirteen CpG sites, though CpG sites are primarily concentrated in the P3 region. Within the P2 region, the five upstream CpG sites are widely spaced, whereas the remaining eight sites are more clustered (Figure 4D, blue bold line). To examine methylation status of the region containing the 8 CpG sites, methylation-specific sequencing (MSS) was performed. Seventeen individual DNA clones from E-stage (DT2) and M-stage (DT5) HDFs undergoing RS revealed that four CpG sites (−445, −366, −363, and −358) within the P2 region (indicated by a green triangle in Figure 4D) were highly methylated in E-stage HDF (overall 54.4%; site 1, 52.9%; site 2, 58.8%; site 3, 52.9%; site 4, 52.9%) but became demethylated in M-stage HDF (Figure 4E). Methylation-specific PCR analysis (MSP) further confirmed this dynamic regulation, showing an obvious decrease in methylation level and an increase in unmethylation level at these four CpG sites during the E-M-A progression of RS, peaking at DT5 (Figure 4F). DNMT1 knockdown in E-stage HDF (DT2) resulted in a similar decrease in methylation level and an increase in unmethylation level at these CpG sites (Figure 4G). These results establish that DNMT1 regulates *SPINT2* transcription via DNA methylation at specific CpG sites, and this epigenetic mechanism contributes to the initiation of HDF senescence.

### SPINT2 suppresses c-Met signaling to induce senescence

**Figure 5:**
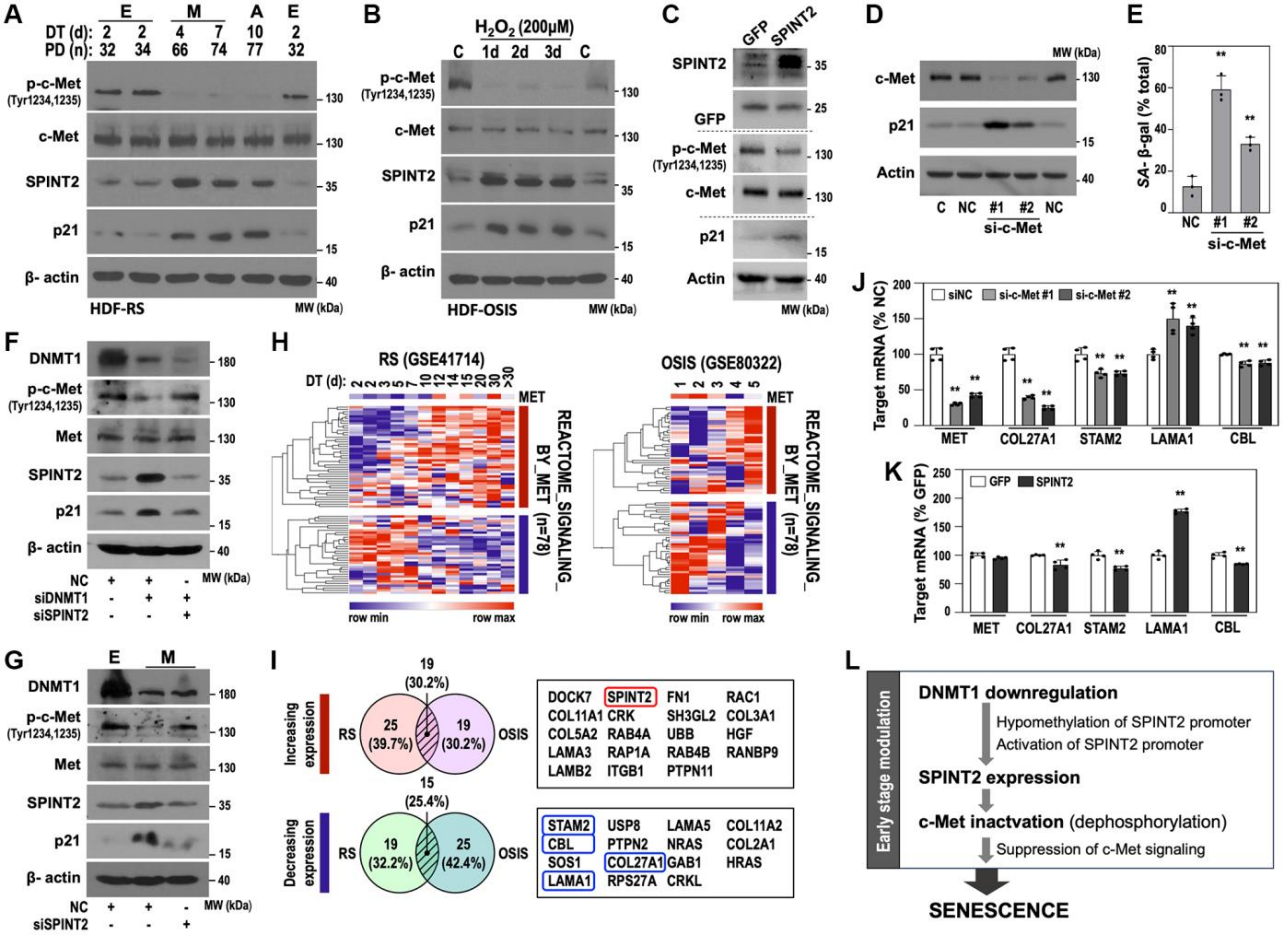
**SPINT2 induces senescence by inhibiting c-Met signaling.** (**A**) Western blot analysis of phosphorylated c-Met (p-c-Met), total c-Met, SPINT2, and p21 protein expression in the RS model HDFs. (**B**) Western blot analysis of p-c-Met, c-Met, SPINT2, and p21 in the time-series OSIS model. (**C**) HDFs (DT2) were infected with recombinant lentivirus encoding *SPINT2* cDNA for 4 days, followed by Western blot analysis. GFP-encoded recombinant lentivirus was used as a control. (D-E) HDFs (DT2) were transfected with siRNAs targeting negative control (NC) or c-Met (si-c-Met) for 4 days. (**D**) Western blot analysis of DNMT1, SPINT2, and p21 protein expression. (**E**) Quantification of SA-β-gal activity. ^**^p < 0.01 vs. NC by Student’s *t*-test. (**F**) HDFs (DT2) were transfected with siRNAs targeting DNMT1 (siDNMT1) and/or SPINT2 (siSPINT2) for 4 days. Western blot analysis was performed. (**G**) M-stage HDFs (DT4) were transfected with siRNAs targeting SPINT2 (siSPINT2) for 4 days. E-stage HDFs were also used as control. Western blot analysis. (**H**) Heatmap presentation of differentially expressed c-Met signaling genes based on transcriptomic data from RS and OSIS models (GSE41714 and GSE80332, respectively). (**I**) Venn diagram depicting the overlap in deregulated c-Met signaling genes between the RS and OSIS models. Among these, 15 genes were commonly downregulated, and 19 genes were commonly upregulated. (**J**) HDFs (DT2) were transfected with c-Met siRNA (si-c-Met) for four days, followed by qRT-PCR analysis of c-Met target gene expression. ^**^p < 0.01 vs. NC by Student’s *t*-test. (**K**) HDFs (DT2) were infected with recombinant lentivirus encoding *SPINT2* cDNA for 4 days, followed by qRT-PCR analysis of c-Met target genes. GFP-encoded recombinant lentivirus was used as a control. ^**^p < 0.01 vs. GFP by Student’s *t*-test. (**L**) Schematic diagram of early senescence modulation.

Since *SPINT2* is known to modulate HGF/c-Met signaling [23, 29, 30], we investigated whether c-Met inhibition is involved in *SPINT2*-mediated senescence. During RS progression, c-Met phosphorylation on Tyr 1234/1235 (activated form) [30] significantly declined from the M stage onward, without altering total c-Met expression, while SPINT2 expression increased (Figure 5A). A similar pattern was observed in OSIS, starting from day 1 (Figure 5B). *SPINT2* overexpression reduced c-Met phosphorylation, but not total c-Met protein levels, suggesting that *SPINT2* inactivates c-Met signaling rather than reducing c-Met expression (Figure 5C). Moreover, c-Met knockdown alone was sufficient to induce senescence phenotypes, including p21 induction and increased SA-β-gal activity (Figure 5D, 5E). DNMT1 knockdown clearly suppressed c-Met phosphorylation and additional SPINT2 knockdown restored the phosphorylation (Figure 5F). Decreased c-Met phosphorylation status of the M stage HDF was also restored by SPINT2 knockdown (Figure 5G). These results clearly explain that SPINT2 is a key mediator of DNMT1-mediated c-Met inhibition in early transition of HDF senescence.

Transcriptomic analysis of c-Met signaling genes in RS (GSE41714) and OSIS (GSE80322) revealed 19 commonly upregulated and 15 commonly downregulated genes (Figure 5H, 5I). Among the downregulated c-Met target genes, significantly suppressed expression of *COL27A1*, *STAM2*, and *CBL*, but not *LAMA1* [31–34], was validated in c-Met-knockdown HDF (Figure 5J). *SPINT2* overexpression replicated a similar pattern (Figure 5K). These findings confirm that DNMT1-mediated SPINT2 modulation drives HDF senescence via c-Met signaling inhibition (Figure 5L).

## DISCUSSION

Cellular senescence is a fundamental biological process that contributes not only to organismal aging but also to the pathogenesis of a wide range of aging-associated diseases, including cancer and metabolic disorders [35]. Therapeutic strategies that specifically target senescent cells have emerged as promising strategies for mitigating or treating such senescence-associated diseases [35, 36]. However, most current strategies predominantly focus on the selective clearance of fully senescent cells, ignoring the early senescent cells and their potential threat. This strategic limitation is largely due to the lack of reliable biomarkers for cells in the early stage of senescence.

In this study, we identified an inverse relationship between DNMT1 and SPINT2 as a critical event for the early transition to senescence. Transcriptomic analysis showed that *DNMT1* levels progressively declined, while *SPINT2* expression increased at the early stage of senescence, preceding SA-β-gal activation. Functional studies confirmed that DNMT1 suppression by siRNA or pharmacological inhibition induced SPINT2-dependent senescence, evidenced by SPINT2 knockdown rescued this phenotype. Although previous studies have reported hypermethylation of *SPINT2* and its tumor-suppressive effects in glioblastoma [37], its role in senescence and direct regulation by DNMT1 were unknown. Our findings show that DNMT1 directly regulates *SPINT2* transcription by targeting four CpG sites (−445, −366, −363, and −358 bp from transcription start) in the *SPINT2* promoter. Loss of *DNMT1* leads to persistent hypomethylation at these sites, resulting in sustained *SPINT2* upregulation and senescence initiation. While the involvement of DNMT1 in regulating senescence-associated genes such as TP53 and Wnt5A has been previously documented [9, 19], this study is the first to establish SPINT2 as a direct downstream effector of DNMT1, providing novel insights into the molecular mechanisms that initiate cellular senescence.

SPINT2 is a well-known inhibitor of HGFA, matriptase, and other extracellular proteases [24]. Among these targets, HGFA is the key activator of the HGF/c-Met pathway [24], which regulates cell survival and proliferation [25, 26]. Given that c-Met inhibition induces senescence-like phenotypes in cancer cells [27, 38], we hypothesized that SPINT2 promotes senescence through HGF/c-Met suppression. Our results strongly support this hypothesis: First, SPINT2 overexpression reduced c-Met phosphorylation, but not total c-Met levels. Second, c-Met knockdown induced senescence phenotypes, mimicking SPINT2 overexpression. Third, transcriptomic analysis identified 19 commonly downregulated c-Met signaling genes in RS and OSIS models, with *COL27A1*, *STAM2*, and *CBL* validated as downstream targets of SPINT2-mediated c-Met suppression. These findings indicate that SPINT2-mediated c-Met inhibition is a fundamental mechanism driving senescence.

Our study provides key insights into the epigenetic regulation of senescence and identifies DNMT1-mediated SPINT2 expression as a novel regulatory axis in early senescence. Since cellular senescence is involved in aging, tissue degeneration, and cancer, modulating DNMT1, SPINT2, and c-Met signaling could provide novel therapeutic opportunities. In aging-related diseases, preventing DNMT1 loss or inhibiting SPINT2 may delay senescence and maintain cellular function. Conversely, in cancer therapy, inducing SPINT2-mediated c-Met suppression could promote tumor cell senescence, limiting proliferation. Future research is needed to determine the broader implications of the DNMT1-SPINT2-c-Met axis in different tissues and disease contexts.

In summary, our study establishes *DNMT1* downregulation as an early molecular event in HDF senescence and identifies SPINT2 as a key effector in this process. We demonstrate that SPINT2 expression is regulated by DNMT1-mediated DNA methylation and its upregulation leads to the suppression of c-Met signaling, ultimately driving senescence-associated phenotypes. These findings uncover a novel epigenetic mechanism governing senescence initiation and offer potential targets for therapeutic intervention in aging and senescence-associated disorders. Further investigations are needed to explore the role of this regulatory axis in different biological contexts and its potential clinical applications in aging and aging-associated disorders.

## MATERIALS AND METHODS

### Cell culture and development of senescence

Primary human dermal fibroblast (HDF) isolated from the foreskin of a 5-year-old boy [4] were used in this study. HDF was cultured in DMEM supplemented with 10% FBS and 1% Antibiotic-Antimycotic (Gibco Life Technologies, Grand Island, NY, USA) at 37°C in a humidified atmosphere containing 5% CO_2_. To develop replicative senescence (RS) of HDFs, confluent cells were continuously subcultured by evenly transferring them into two new dishes. The numbers of population doubling (PD) and the doubling time (DT) were monitored, as previously described [4]. It is noteworthy that DT includes the time period for cells to adapt for growing after even splitting of cell population from a culture dish. To generate oxidative stress-induced senescence (OSIS), the primary HDF with a doubling time of 2 days (DT2) were treated with subcytotoxic doses (100–300 μM) H_2_O_2_ twice, with a 12 h interval between the treatments. Then, the cells were further incubated for the indicated time periods, as described previously [9].

### Senescence-associated β-galactosidase activity (SA-β-gal) staining

SA-β-gal assay was carried out using the Senescence β-Galactosidase Staining Kit (Cell Signaling Technology, Beverly, MA, USA) according to the manufacturer’s protocol. The percentage of senescent cells was estimated by counting the number of blue-stained cells and total cells using ImageJ Software (NIH, Bethesda, MD, USA).

### Estimation of cell size and cellular granularity

To estimate cell size and granularity, the cells were stained with MitoTracker Red CMX ROS (Molecular Probes, Eugene, OR, USA) for 15 minutes at 37°C prior to flow cytometric analysis using the FACS Canto^™^ II instrument (Becton Dickinson Corp., San Jose, CA, USA). The cell size and cellular granularity of 10,000 stained cells were evaluated by analyzing the forward scattering (FSC) and side scattering (SSC), respectively, as previously described [4].

### Introduction of siRNAs into cells

HDFs were transfected with siRNA duplexes and plasmids using Lipofectamine RNAiMAX^™^ reagent (Invitrogen Life Technologies, Carlsbad, CA, USA) and FuGENE HD (Roche Diagnostics), respectively, following the manufacturer’s instructions. All siRNAs for targets, *DNMT1* (#1, 5′-CGAGUCUGGUUUGAGAGUTT; #2, 5′-UCUGUCCGUUCACAUGUGTT), *SPINT2* (5′-CAGAAGGCAGGAUUCUGAATT), *c-Met* (#1, 5′-GUGAAGAUCCCAUUGUCUATT; #2, 5′-GAGUUCUCCUUGGAAAUGATT) and Negative control (NC, 5′-CCUACGCCACCAAUUUCGUTT) were obtained by Bioneer (Daejeon, Korea).

### Generation of recombinant SPINT2 lentivirus and its infection into cells

To generate a recombinant lentiviral plasmid containing *SPINT2* cDNA (pLenti-GIII-CMV-SPINT2-GFP-2A-puro), *SPINT2* cDNA was amplified using total cDNA of the primary HDF and a primer set (5′-CCCGATATCATGGCGCAGCTGTGC and 5′-CCCGATATCTCACAGGACATATG) and inserted into pLenti-GIII-CMV-GFP-2A-puro plasmid (Applied biological Materials, Richmond, BC, CANADA) using EcoV restriction enzyme. To generate recombinant lentiviral particles, HEK-293TN packaging cells (ATCC, Rockville, MD, USA) were cultured in DMEM and transfected with the recombinant lentiviral plasmids using Lipofectamine^™^ 2000 reagent (Invitrogen Life Technologies, Carlsbad, CA, USA). The virus-containing medium was harvested daily for 4 days, filtered through a 0.45-μm filter unit (EMD Millipore Corp. Darmstadt, Germany), and stored at −80°C. A filtered medium containing recombinant lentiviral particles was mixed with polybrene (8 μg/ml, Sigma-Aldrich, Burlington, MA, USA) for facilitated infection. After the infection with the recombinant lentivirus, cells were incubated for 12 hours and refreshed with a fresh medium for 3 days. pLenti-GIII-CMV-GFP-2A-puro (Applied Biological Materials, Richmond, BC, Canada) alone was also used as an infection and expression control.

### Construction of the promoter-luciferase reporter plasmid and promoter assay

A promoter region (−1700 to +300 from the transcription start, NC_000019.10) of human SPINT2 gene was cloned by performing targeted PCR against total genomic DNA extracted from HDFs, using a primer set, 5′-TATGAGCTCCTCTGTTTTCATGCTCAAGCC and 5′-TGCAGATCTCTGCGGACATCGTCG. The amplified SPINT2 promoter region was inserted between the SacI and NheI sites of the pGL4-basic vector (Promega, Fitchburg, WI, USA), generating the pGL4-SPINT2 promoter reporter plasmid (pGL4-SPINT2 promoter). Three additional deleted forms of SPINT2 promoter-reporter plasmids, ΔP1 (deletion of −1700 to −839), ΔP1+ΔP2 (deletion of −1700 to −268), ΔP2 (deletion of −839 to −268) were constructed by using paired restriction enzymes, Eco53kI/BSAaI, Eco53kI/ZraI and BSAI/ZraI, respectively. After construction, the accuracy of all the inserted promoter regions was confirmed by DNA sequencing.

To monitor SPINT2 promoter activity, cells were transfected with a total of 1 μg of DNA (950 ng of the cloned reporter plasmid and 50 ng of thymidine kinase promoter-driven renilla luciferase plasmid, an internal assay control) using FuGENE HD reagent (Promega, Madison, WI, USA). After 4 days, the luciferase activity of cell extracts was measured by Synergy 2 Multi-Mode Reader (BioTek Instruments, Inc., Winooski, VT, USA) according to the protocol provided with the Dual-Luciferase Reporter Assay System (Promega, Madison, WI, USA). The firefly luciferase activities were normalized to the renilla luciferase activity.

### DNA methylation-specific sequencing and methylation-specific PCR

Potential CpG islands within the SPINT2 promoter region (−1700 to +300 bp from the transcription start, NC_000019.10) were estimated using the MetPrimer program. To determine the methylation pattern of the cell, DNA methylation-specific sequencing (MSS) was performed as previously reported [9]. Briefly, total genomic DNA (500 ng) isolated from conditioned HDF was modified with sodium bisulfite according to the instruction of the EZ DNA methylation kit (Zymo Research, Orange, CA, USA). The bisulfite-treated DNA was used as a template for PCR amplification of a targeted promoter region (−474 to −270 bp of SPINT2 promoter) using the following primer set, 5′-TGATTTAGGGATTAGTTTGGTATGG and 5′-CCACCCTTCCCTTAAACTTAATAAC (Cosmogenetech Inc. Seoul, Korea). The PCR product was subcloned into the pGEM T-easy vector (Promega, Madison, WI, USA). Multiple clones (17 clones) were selected using 50 μg/ml Ampicillin (Sigma-Aldrich, St. Louis, MO, USA) and isolated gDNA clones were subjected to DNA sequencing at Cosmogenetech Inc. (Seoul, Korea).

To perform methylation-specific PCR (MSP), the CpG-rich region containing four methylation hot spots (−445, −366, −363, and −358 bp upstream of the transcription start) which were identified by MSS was amplified against the bisulfite-modified DNA using the following two primer sets (Cosmogenetech Inc. Seoul, Korea). The primer set (−469 to −333 bp, 137 bps) to detect methylated status was 5′-TAGGGATTAGTTTGGTATGGTTATC and 5′-CTTAAAAACCTATAATTTTCTACCCG. The primer set (−467 to −336 bp, 132 bps) for the unmethylated status was 5′-GGGATTTTGGTATGGTTATTGT and 5′-AAAAACCTATAATTTTCTACCCACC. PCR condition was as follows: 95°C for 5 min, followed by 40 cycles of 95°C for 30 s, additional cycles of 56°C for 30 s and 72°C for 1 min and a final extension at 72°C for 10 min.

### Western blotting analysis

Western blotting analysis was performed using standard procedures. Antibodies against DNMT1 (GTX116011) and SPINT2 (GTX52776) were purchased from GeneTEX (Irvine, CA, USA). Antibodies against p21 (sc-6246) and β-actin (sc47778) were obtained from Santa Cruz Biotechnology (Dallas, TX, USA), and the antibody for Turbo GFP (TA150041) from Origene (Rockville, MD, USA). Antibodies against p-Met (Tyr1234/1235, #3077) and c-Met (#3127) were purchased from Cell Signaling Technology (Beverly, MA, USA). All antibodies were used at a 1:1000 dilution except β-actin which was used at 1:5000 dilution.

### RNA extraction and quantitative RT-PCR

Total RNA was isolated using TRIzol (Invitrogen Life Technologies, Carlsbad, CA, USA), and cDNA was prepared using avian myeloblastosis virus (AMV) reverse transcriptase (Promega, Madison, WI, USA). PCR was performed using Go Taq qPCR Master Mix (Promega, Madison, WI, USA) according to the protocol of the manufacturer. The primer sets were produced by Bioneer (Daejeon, Korea) as follows: DNMT1, 5′-TACCTGGACGACCCTGACCTC and 5′-CGTTGGCATCAAAGATGGACA; SPINT2, 5′-GATTCCTCTGTCCCAAGTGC and 5′-TATCGCTGGAGTGGTCTTCA; Met, 5′-TGCAGCGCGTTGACTTATTC and 5′-GAAACCACAACCTGCATGAAG; TBP, 5′-CACCTTACGCTCAGGGCTT and 5′-CTGAATAGGCTGTGGGGTCA; COL27A1, 5′-AATGGCAGGTCTCTTCGGAC and 5′-GGACCTTGCAATCCCCAGTC; STAM2, 5′-GCGAGATGCCTTTGTTCACC and 5′-GGCAATCTTTCGCTCCATTAGG; LAMA1, 5′-CGTGAGGACCCCAGTAACG and 5′-CAGTAAAACGGCTCTGCACG; CBL, 5′-CTCATGGACAAGGTGGTGCG and 5′-AGTACGGAGATGCTGGTAGG: β-actin, 5′-CCTTCCTGGGCATGGAGTCCTGT and 5′-GGAGCAATGATCTTGATCTTC. The relative expression of target genes was calculated using the 2−ΔΔCt method [39] against β-actin expression as the internal control.

### Transcriptomic analysis of public datasets from cellular senescence models

Transcriptome data from senescence models (RS and OSIS) of human primary fibroblasts, previously generated by our group [4, 9], were retrieved from the NCBI Gene Expression Omnibus (GEO) under accession codes GSE41714 and GSE80322, respectively. Probe IDs were mapped to gene symbols using the mapIds function in the R Annotation Dbi package (v1.52.0). Morpheus (https://software.broadinstitute.org/morpheus/) was employed to generate expression heatmaps of selected genes and gene sets and to perform hierarchical clustering using one minus Pearson correlation distance and average linkage. Correlation analysis was performed using the cor.test function in the R stats package (v.4.2.1) to compute Pearson correlation coefficient and *p*-values.

### Statistical analysis

Statistical analyses of transcriptomes were performed using R (v.4.2.1) as described above. All experiments were repeated at least three times. Data were presented as mean ± standard deviation. Statistical analysis was performed using unpaired two-samples Student’s *t*-test using GraphPad Prism software (La Jolla, CA, USA).

### Data availability

Microarray datasets analyzed in this study can be found at the NCBI GEO under the accession codes GSE41714 and GSE80322.
